# Detection and genomic characterization of West Nile virus lineage 2 in captive harbor seals in southern Italy

**DOI:** 10.3389/fmicb.2026.1937304

**Published:** 2026-09-16

**Authors:** Claudio de Martinis, Lorena Cardillo, Marta Giovanetti, Alessia Pucciarelli, Gerardo Picazio, Maurizio Viscardi, Loredana Cozzolino, Sara Cavaliere, Alessia Napolitano, Maria Lella, Maria Dimatteo, Barbara Degli Uberti, Pasquale Silvestre, Evaristo Di Napoli, Nicola D’Alessio, Esterina De Carlo, WNV One Health Surveillance Network, Giorgio Galiero, Giuseppe Iovane, Orlando Paciello, Giovanna Fusco

**Affiliations:** 1 Department of Animal Health, Istituto Zooprofilattico Sperimentale del Mezzogiorno, Naples, Italy; 2 Department of Science and Bio-Technology, Università Campus Bio-Medico di Roma, Rome, Italy; 3 Oswaldo Cruz Institute, Oswaldo Cruz Foundation (Fiocruz), Rio de Janeiro, Brazil; 4 Azienda Sanitaria Locale Napoli 2 Nord, Naples, Italy; 5 Lo Zoo di Napoli, Naples, Italy; 6 Department of Veterinary Medicine and Animal Production, University of Naples "Federico II", Naples, Italy; 7 National Reference Centre for Hygiene and Technologies of Water Buffalo Farming and Productions, Istituto Zooprofilattico Sperimentale del Mezzogiorno, Salerno, Italy; 8 General Direction, Istituto Zooprofilattico Sperimentale del Mezzogiorno, Portici, Naples, Italy; 9 Sanitary Direction, Istituto Zooprofilattico Sperimentale del Mezzogiorno, Portici, Naples, Italy; 10 Caserta Section, Istituto Zooprofilattico Sperimentale del Mezzogiorno, Tuoro, Caserta, Italy

**Keywords:** fatal cases, genetic characterization, harbor seals, lineage 2, West Nile virus

## Abstract

**Introduction:**

West Nile virus (WNV) is a mosquito-borne flavivirus maintained in bird–mosquito cycles, with occasional spillover events causing neuroinvasive disease in humans and animals. This study describes two fatal cases of WNV lineage 2 infection in captive harbor seals housed in a zoological park in southern Italy, together with genomic characterization and phylogenetic analysis of the detected strains.

**Methods:**

Harbor seals were tested using multiplex real-time reverse transcription-polymerase chain reaction (RT-PCR) for the simultaneous detection of WNV lineage 1 and lineage 2. Selected extracts were further analyzed by droplet digital PCR (ddPCR). Next-generation sequencing was performed, and the resulting sequences were subjected to phylogenetic analysis. A Centers for Disease Control and Prevention (CDC) light trap was used for targeted mosquito collection near the fence of the dead harbor seals, and female mosquitoes were screened for WNV. Histopathological examination was also performed in one seal.

**Results:**

WNV lineage 2 RNA was detected in central nervous system tissues of both seals and in a pool of female *Culex pipiens* collected near the fence. Near-complete WNV genomes clustered with contemporary southern Italian strains and were consistent with local viral circulation.

**Discussion:**

These findings document WNV lineage 2 infection associated with fatal neurological disease in harbor seals, expanding current knowledge of the host range of this virus and providing complementary *in situ* entomological and genomic evidence. Overall, this study highlights the value of an integrated One Health surveillance approach in zoological parks.

## 1 Introduction

Rapid urban expansion, land-use change, biodiversity loss, and climate-driven shifts in vector ecology are increasingly associated with the emergence and re-emergence of vector-borne diseases worldwide (Singh et al., 2025). Among these, West Nile virus (WNV) has become a major public and veterinary health concern in several regions, including Europe. WNV is an enveloped, positive-sense, single-stranded RNA arbovirus belonging to the family *Flaviviridae*. The virus is primarily maintained through enzootic transmission cycles involving wild birds and mosquitoes of the genus *Culex*, while humans and other vertebrates generally act as incidental dead-end hosts (Petersen et al., 2013; Chancey et al., 2015).

Among the recognized WNV lineages, lineages 1 and 2 are most commonly associated with clinically relevant disease in humans and animals (Odigie et al., 2024; Center for Food Security and Public Health, 2026). Since its introduction into Europe, lineage 2 has progressively replaced lineage 1 in many regions and is currently responsible for most reported human and animal cases (Barzon et al., 2013; Savini et al., 2013). Over the last decade, WNV has become firmly established in several European countries, including Italy, where seasonal outbreaks are regularly documented (Riccardo et al., 2022). In Europe, *Culex pipiens* is considered the principal vector sustaining transmission, although WNV has also been detected in several other mosquito taxa (Varga et al., 2024).

Clinical manifestations of WNV infection range from asymptomatic or mild febrile illness to severe neuroinvasive diseases, including meningitis, encephalitis, and acute flaccid paralysis, which represent the most clinically significant forms of infection in humans and several animal species, particularly equids and some avian hosts (Petersen et al., 2013; Chancey et al., 2015).

Although birds remain the principal amplifying hosts, WNV has been detected in a broad range of mammalian species (Root and Bosco-Lauth, 2019; Vilibic-Cavlek et al., 2021; Simonin, 2024; Byas et al., 2022), highlighting the ecological complexity of the virus at the wildlife–vector interface. Field investigations and experimental studies have shown that some wild mammals can develop measurable viremia following infection and, in selected species, viremia levels may theoretically be sufficient to contribute to mosquito infection under specific conditions (Root and Bosco-Lauth, 2019). Reports of infection or seropositivity in atypical hosts, such as dromedaries and opossums, further illustrate the extensive host range of WNV, while disease outbreaks and mortality events associated with WNV infection have also been documented in American alligators (Root and Bosco-Lauth, 2019; Byas et al., 2022). In addition, a growing number of mammalian species have tested positive for WNV, providing opportunities to investigate viral dynamics at the human–animal–vector interface (Root and Bosco-Lauth, 2019; Vilibic-Cavlek et al., 2021; Simonin, 2024; Byas et al., 2022).

Despite this expanding evidence, reports of WNV infection in marine mammals remain exceptional (Del Piero et al., 2006). Collectively, these observations indicate that several aspects of WNV ecology remain incompletely understood, particularly regarding atypical hosts and the circumstances under which they may contribute to local transmission dynamics.

This study describes WNV lineage 2 infection in two captive harbor seals (*Phoca vitulina*) housed in a zoological park in the Campania region (Naples, southern Italy) and presents an integrated One Health investigation combining animal diagnostics, mosquito surveillance, and genomic characterization.

## 2 Materials and methods

### 2.1 Sample collection and general analysis

Two captive harbor seals (*Phoca vitulina*) housed at the zoological park “Lo Zoo di Napoli” (Naples, southern Italy) were included in this study (Figure 1a). Individual identification and origin data were obtained from institutional records. Seal 1 and Seal 2 were two male harbor seals aged 3 and 2 years, respectively. Both individuals were born at the Frankfurt Zoo and transferred to Naples in November 2024, showing good general condition.

**Figure 1:**
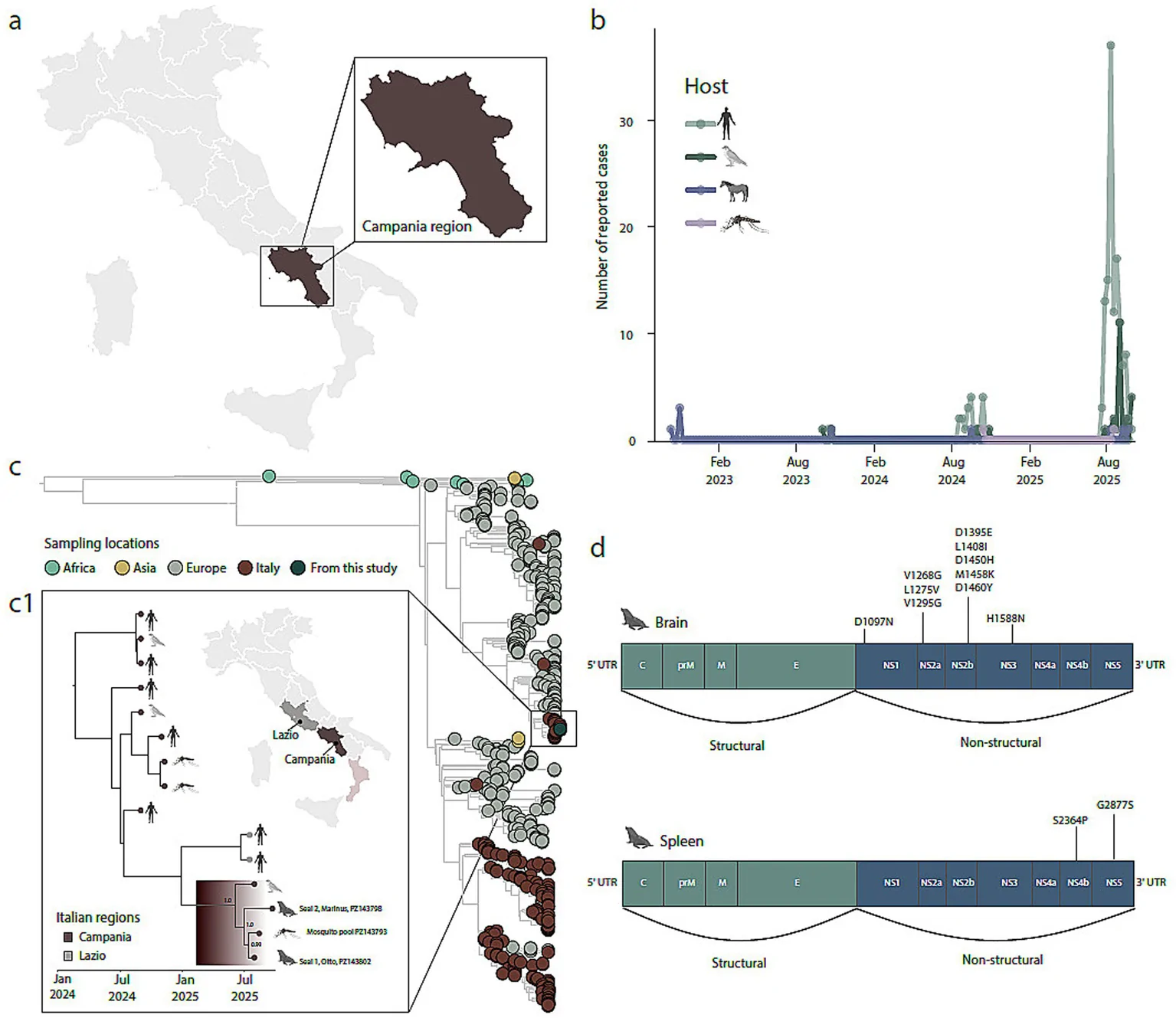
Detection and genomic characterization of West Nile virus lineage 2 in captive seals, Campania Region, Southern Italy, 2025. **(a)** Geographic location of the zoological park in the Campania Region of Southern Italy where two captive harbor seals tested positive for WNV. **(b)** Temporal distribution of reported WNV cases in Italy by host category (humans, birds, mammals, and mosquitoes) during 2023–2025, highlighting the contemporaneous increase in activity in 2025. **(c)** Maximum-likelihood phylogeny of WNV lineage 2 genomes including the seal-derived sequences generated in this study (highlighted), showing clustering within a recent Southern Italian transmission clade. The inset represents the regional distribution of related Italian genomes. **(d)** Schematic representation of the WNV polyprotein indicating the location of synonymous and missense substitutions identified in brain- and spleen-derived genomes, with mutations predominantly affecting non-structural proteins.

Routine husbandry observations and veterinary clinical assessments were performed, with additional examinations and sampling performed during episodes of illness. Whole blood and serum were collected for hematology and serum biochemistry. Complete blood counts, biochemical profiles, and serum protein electrophoresis, as well as a rapid immunochromatographic assay for the detection of anti-*Toxoplasma gondii* immunoglobulin M/immunoglobulin G (IgM/IgG) antibodies for Seal 1, were performed by an external veterinary laboratory (LABforVET, Bagnoli, Naples, Italy).

Furthermore, Seal 1 underwent nasal swab administration. Bacterial culture and identification were performed, followed by antimicrobial susceptibility testing, and the minimum inhibitory concentration (MIC) was determined to obtain categorical results classified as susceptible, intermediate, or resistant. Furthermore, swab samples were screened for selected respiratory pathogens by real-time RT-PCR for the detection of canine parainfluenza virus (CPIV), canine respiratory coronavirus (CRCoV), canine distemper virus (CDV), and canine pneumovirus (CnPnV) and by real-time PCR for the detection of canine adenovirus type 2 (CAV-2), *Mycoplasma cynos*, and *Bordetella bronchiseptica*.

At death, the carcasses were submitted to the Istituto Zooprofilattico Sperimentale del Mezzogiorno (IZSM) to establish the cause of death. A complete necropsy was therefore performed by postgraduate veterinarians, followed by the collection of brain, heart, spleen, kidneys, lungs, trachea, submandibular lymph nodes, and testes samples for virological, bacterial, and protozoan investigations. Details of all investigated pathogens and protocols are reported in Supplementary Table 1.

### 2.2 West Nile virus and Usutu virus biomolecular and serological analyses

Brain, heart, spleen, and kidney tissues of the two harbor seals, together with whole blood, were transferred to the Unit of Exotic and Vector-borne Diseases of IZSM and processed in a Biosafety Level 3 (BSL-3) laboratory. Tissue homogenization, nucleic acid extraction, and real-time RT-PCR for the detection of WNV lineages 1 and 2 and Usutu virus (USUV) were performed as reported by de Martinis et al. (2023a), using protocols previously described (Del Amo et al., 2013; Cavrini et al., 2011). Positive samples were confirmed by the National Reference Centre for Exotic Animal Diseases (Centro di Referenza Nazionale per le malattie esotiche degli animali – CESME, Istituto Zooprofilattico Sperimentale dell’Abruzzo e del Molise, Teramo, Italy).

Additionally, WNV-positive samples were analyzed by droplet digital PCR (dd-PCR) in duplicate for absolute viral quantification, as previously described (de Martinis et al., 2023b), using the same real-time RT-PCR primers and probe sequences (Del Amo et al., 2013). PCR amplification was conducted on a T100 Thermal Cycler (Bio-Rad Laboratories, Hercules, CA, USA) with the following thermal profile: reverse transcription at 50 °C for 60 s, initial denaturation at 95 °C for 10 min, followed by 45 cycles at 95 °C for 30 s and 60 °C for 60 s, one cycle at 98 °C for 10 min, and a final hold at 4 °C. Samples were considered positive when at least three positive droplets were detected, in accordance with the manufacturer’s instructions.

For genomic characterization, RNA extracts with sufficient viral signal were selected for next-generation sequencing (NGS), as previously described (de Martinis et al., 2023a), and run on the MiSeq platform (Illumina, San Diego, CA, United States) using the v2 Reagent Cartridge, with 300 cycles and 150 bp paired-end reads. FASTQ files were generated with BaseSpace Sequence Hub (Illumina) and assembled using Geneious R9.13.

Finally, a serum sample from Seal 2 was analyzed to evaluate the presence of anti-WNV IgG antibodies. The serum was obtained by centrifugation at 1,300 × *g* for 10 min (Centrifuge 5,810, Eppendorf, Hamburg, Germany) and tested using the ID Screen Flavivirus Competition Multi-species (IDvet, Grabels, France) according to the manufacturer’s instructions. The sample was then transferred to CESME to confirm ELISA positivity using specific WNV and USUV serum neutralization assays.

Following the detection of WNV RNA in the carcasses, an entomological investigation was implemented to assess local viral circulation in the zoo environment. A CDC light trap was placed near the seal fence for overnight collection. Mosquitoes were morphologically identified to the genus or species level by stereomicroscopy, and taxonomic identification was performed using identification keys implemented in MosKeyTool. The mosquitoes were then pooled and analyzed for WNV and USUV, as described above.

### 2.3 Phylogenetic analysis

Newly generated genomes underwent phylogenetic analysis, including representative WNV lineage 2 sequences from Italy and Europe (*n* = 524) retrieved from public databases. Sequences were aligned with the multiple alignment using fast Fourier transform algorithm (MAFFT; Katoh et al., 2019) and inspected in AliView (Larsson, 2014). Maximum-likelihood phylogenies were inferred with IQ-TREE under an appropriate nucleotide substitution model (Nguyen et al., 2015). Temporal signal was assessed with TempEst (Rambaut et al., 2016), and time-scaled reconstructions were performed in BEAST using a relaxed molecular clock and Skygrid coalescent prior (Suchard et al., 2018).

### 2.4 Histopathological examinations

Representative samples from the brain, spinal cord, bladder, heart, lung, and small intestine were fixed in 10% neutral-buffered formalin for 24 h, routinely processed, and embedded in paraffin. Serial sections, 3–4 μm thick, were obtained from each sample and stained with hematoxylin and eosin (H&E) with a standardized protocol (De Biase et al., 2024). Slides were initially examined under light microscopy for morphological assessment and subsequently digitized using a Pannoramic 250 Flash III slide scanner (3DHISTECH, Budapest, Hungary), acquiring whole-slide images at reference magnifications of 6x, 10x, and 100x.

## 3 Results

By the end of July 2025, Seal 1 developed afternoon anorexia without any evidence of general malaise. On the following day, kyphosis was observed, prompting the capture of the animal for clinical examination. Blood samples, two nasal swabs for virological and bacteriological analyses, and a cloacal swab were collected. No abnormalities were detected in the vital parameters. Subsequently, the animal exhibited rapid and progressive clinical deterioration, characterized initially by inspiratory crackles and later by impaired swimming ability associated with paralysis of the caudal and right pectoral flippers.

Initial hematological and biochemical analyses were performed (Supplementary material). Molecular assays and serological testing for anti-*Toxoplasma gondii* IgM and IgG antibodies were negative, whereas *Pseudomonas aeruginosa* was isolated from a nasal swab. Based on the results of minimum inhibitory concentration (MIC) antimicrobial susceptibility testing, treatment with enrofloxacin was initiated, together with a non-steroidal anti-inflammatory drug and vitamin B supplementation.

Despite therapeutic intervention, the clinical condition continued to deteriorate over the following days. By day 4 after the onset of clinical signs, the seal developed severe neurological manifestations, such as depression, opisthotonos, nuchal rigidity, and respiratory distress. The therapeutic protocol was therefore intensified by adding a cephalosporin, and an electrolyte rehydration solution was administered. Nevertheless, 6 days after the onset of the initial clinical signs, the animal developed near-complete truncal paralysis, persistent opisthotonos, and nystagmus. Given the poor prognosis and progressive neurological deterioration, compassionate euthanasia was performed.

The carcass underwent complete necropsy examination. Body condition was fair, with moderate perianal diarrheic material. Gross findings included reactive maxillary and submandibular lymph nodes, enlargement of the left parotid gland, multifocal congestion and hyperemia involving several organs, including the stomach, liver, kidneys, urinary bladder, trachea, lungs, and brain, as well as mild thoracic and perirenal serohemorrhagic effusions. Pulmonary lesions consisted of multifocal hemorrhagic and emphysematous areas with abundant foamy serohemorrhagic fluid within the airways, while the heart showed a rounded appearance with myocardial dilation. Cerebral examination revealed marked vascular congestion without evident parenchymal alterations. Overall, the gross pathological findings were suggestive of cardiocirculatory arrest associated with an underlying infectious process. Tissue samples were subsequently collected for microbiological and further diagnostic investigations (Supplementary Table 1). The results showed the detection of WNV lineage 2 RNA in the brain (Cycle threshold [Ct] value: 30.8), heart (Ct: 32), and spleen (Ct: 33), confirmed by the National Reference Center (CESME).

Shortly after the death of Seal 1, Seal 2 developed mild anorexia. Vitamin B supplementation was administered as supportive therapy, resulting in the complete resolution of the clinical sign within 3 days. Although no additional clinical abnormalities were observed, serum and EDTA blood samples were collected in mid-August 2025 to investigate the presence of anti-WNV IgG antibodies and WNV RNA, respectively. Both tests yielded positive results, which were subsequently confirmed by the National Reference Centre. Virus neutralization testing (VNT) demonstrated a WNV-specific antibody titer of 1:160.

Despite the absence of overt clinical signs following the initial episode of anorexia, Seal 2 began to exhibit progressive weight loss in mid-September while maintaining a normal appetite. Two days later, anorexia recurred and was accompanied by a rapid deterioration in the animal’s clinical condition. The seal was therefore isolated, and hematological and biochemical analyses were performed (Supplementary material). Shortly thereafter, neurological manifestations consistent with those observed in Seal 1 developed, and the animal died spontaneously the following day.

A necropsy examination was performed, revealing lesions comparable to those described in Seal 1, together with the presence of serohemorrhagic pericardial effusion. Brain (Ct: 34) and spinal cord (Ct: 36) tested positive for WNV lineage 2. Table 1 shows the results of quantitative analysis by dd-PCR.

**Table 1:** Real-time RT-PCR and droplet digital-PCR results.

Species	Organ	Real-time RT-PCR (Ct)	dd-PCR (copies/ μL)	Copies/Reaction	No positive (events)
Seal 1	Brain	30.8	4.55	20.02	77
	Heart	32	5.25	23.1	89
Seal 2	Brain	34	3.85	16.94	60
	Spinal cord	36	1.7	7.48	21
Mosquito	Pool	31.5	3.7	16.28	69

Furthermore, following confirmation of WNV infection in the first animal, an entomological investigation was conducted in the zoo environment. A CDC light trap with CO_2_ generator was placed near the seal enclosure. Collected mosquitoes were morphologically identified as *Aedes albopictus* and *Culex pipiens*. Specimens were processed by taxon, and *Aedes* spp. and *Culex* spp. were handled separately. Only female mosquitoes underwent molecular testing. In total, three *Aedes albopictus* and 21 *Culex pipiens* were processed for virological screening. Real-time PCR testing detected WNV lineage 2 in the *Culex pipiens* females, supporting the presence of infected vectors in proximity to the fence.

NGS generated near-complete WNV genomes from seal tissues and from the WNV-positive mosquito pool [mosquito pool, PZ143793; seal 1, PZ143802; seal 2, PZ143798]. Phylogenetic analysis confirmed that all sequences belonged to lineage 2 and clustered with strains detected in southern Italy during the 2024–2025 transmission seasons, which was consistent with local transmission (Figure 1b, c). A comparative genomic analysis identified both synonymous and missense substitutions across seal-derived genomes, with amino acid changes predominantly located in non-structural proteins [NS1, NS2A, NS3, NS4B, and NS5]. Several substitutions differed between brain- and spleen-derived sequences, suggesting within-host and/or tissue-associated variation (Figure 1d).

Finally, histopathological examinations were also carried out for Seal 2. Leptomeninges, gray matter of the brain, and spinal cord were affected by mild, chronic, multifocal, and perivascular lymphoplasmacytic polioencephalomyelitis (Figure 2). The inflammatory infiltrate was composed of a moderate number of small lymphocytes, few plasma cells, and rare activated macrophages. The leptomeninges were mildly expanded by inflammatory cells and were associated with multifocal congestion. Comparable lesions were also observed in the spinal cord. The lesions were primarily distributed within the gray matter of the mesencephalon and the frontal lobe, where small multifocal foci of gliosis and microglial proliferation were observed. Occasional neuronophagia was present. Inflammation was characterized by mild perivascular lymphoplasmacytic cuffing. Neurons showed degeneration. In addition, similar lymphocytic perivascular cuffing was detected in the epicardium of the left ventricle, associated with mild multifocal interstitial myocarditis and focal cardiomyocyte necrosis. Mild, comparable inflammatory changes were also present in the pulmonary interstitial septa and perivascular spaces. These findings were consistent with viral neuroinvasive disease.

**Figure 2:**
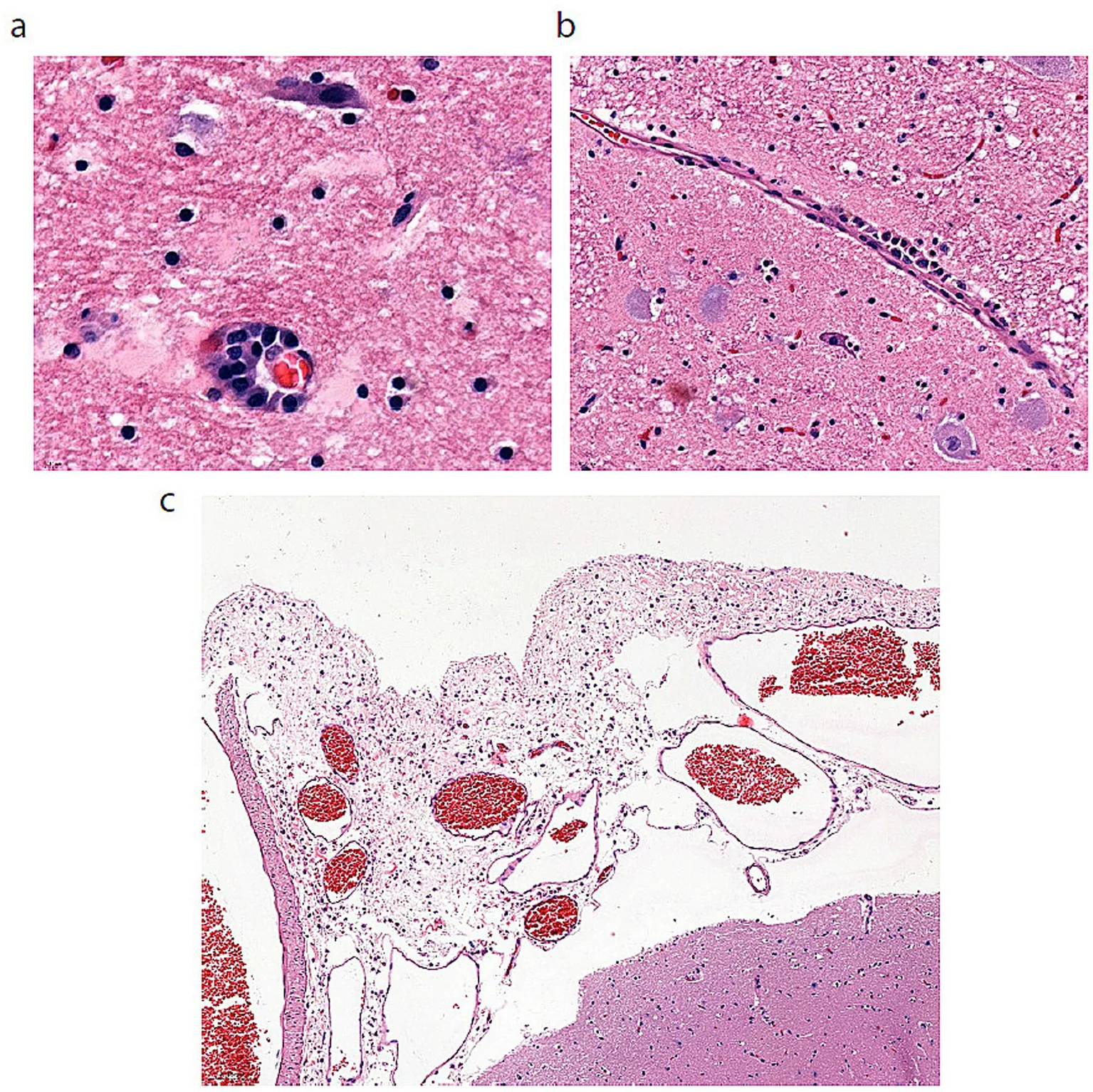
Histopathological findings in WNV-infected harbor seals. **(a,b)** Brain section showing perivascular cuffing predominantly composed of lymphocytes and plasma cells; **(c)** Frontal lobe section demonstrating edematous meninges with ectatic vessels replaced by red blood cells (congestion). Hematoxylin–Eosin staining for **(a–c)**. Original magnification, 100x, 6x, and 10x, respectively. Scale bars, 20 μm.

Overall, the temporal occurrence during the transmission season, the acute neurological syndrome, the detection of WNV lineage 2 RNA in central nervous system tissue in both animals, and the compatible pattern of lymphoplasmacytic polioencephalomyelitis in Seal 2 support a clinicopathological picture consistent with West Nile neuroinvasive infection. Although the observed lesions are not pathognomonic and were mild in severity, they fall within the spectrum of viral encephalomyelitis and, when interpreted alongside the virological findings, support WNV as the most plausible etiology of the neurological disease. The detection of viral RNA in extra-neural tissues, including the spleen in both seals and the heart in Seal 1, further supports systemic infection rather than incidental contamination.

Finally, further carcasses during the same 2025 transmission season and within the same zoological park were referred to the laboratories and submitted to WNV and USUV investigation, and all tested negative. The tested animals are detailed in Table 2. In addition, 10 horses housed in the same location as the tested animals after the detection of the positive seals were serologically tested for the detection of anti-WNV IgM antibodies using ID Screen West Nile IgM Capture (IDvet, Grabels, France), following the manufacturer’s instructions.

**Table 2:** Animal carcasses submitted to the laboratory for necropsy examination and diagnosis.

Species	Scientific name	No of carcasses
Deer	*Dama dama*	5
Geese	*Anser anser*	2
Common pheasants	*Phasianus colchicus*	2
Greater flamingos	*Phoenicopterus roseus*	2
Great white pelican	*Pelecanus onocrotalus*	1
Ouessant sheep	*Ovis aries*	1
Alpaca	*Vicugna pacos*	1
Nyala	*Tragelaphus angasii*	1
Greater rhea	*Rhea americana*	1
Lovebird	*Agapornis* sp.	1
Harris’s hawk	*Parabuteo unicinctus*	1
Lady Amherst’s pheasant	*Chrysolophus amherstiae*	1
Yellow-crowned amazon	*Amazona ochrocephala*	1
Red-lored amazon	*Amazona autumnalis*	1
Goshawk	*Accipiter gentilis*	1
Hawk	*Falco* sp.	1
Duck	*Anas* sp.	1
Total		**24**

The bold value indicates the total number of carcasses. If this formatting is considered unnecessary, the value can be presented in regular font.

## 4 Discussion

This study describes WNV lineage 2 infection in two captive harbor seals in southern Italy, adding to the limited evidence that WNV can occasionally cause clinically overt disease in marine mammals. To date, WNV infection in a harbor seal has been documented only once, in a fatal case reported in North America (Del Piero et al., 2006). Our investigation therefore represents the first detection of WNV in harbor seals in Europe and, importantly, the first report in which this infection is contextualized by contemporaneous *in situ* evidence of viral circulation through entomological surveillance.

The zoological setting is highly relevant to the interpretation of this event. The park hosts a wide variety of vertebrate species, including taxa with known WNV susceptibility and/or a recognized role in the enzootic cycle, particularly birds (Petersen et al., 2013; Chancey et al., 2015). Despite the presence of numerous potentially susceptible zoo animals, all of which tested negative during the same transmission season, clinically overt infection was detected only in harbor seals, underlining how atypical hosts housed within these reconstructed habitats may reveal focal transmission or host-specific vulnerability rather than broad transmission across different animal species (Fukui, 2020). Therefore, the confirmed WNV lineage 2 infection in the harbor seals and the detection of WNV lineage 2 in local *Culex pipiens* are noteworthy. This pattern is consistent with localized exposure but does not allow the identification of the mechanisms responsible for the observed distribution of cases. Several non-mutually exclusive hypotheses may explain these findings, including enclosure-specific ecological conditions, variation in mosquito biting pressure, behavioral factors, or differences in host susceptibility. However, none of these mechanisms were directly investigated in the present study, and they should therefore be interpreted as plausible explanations rather than demonstrated causes. Regardless of the underlying mechanism, these observations highlight that zoological parks can act as interfaces where vector activity, wildlife reservoirs, and susceptible captive hosts overlap and where atypical hosts may represent sentinels of local transmission (Vilibic-Cavlek et al., 2021; Gobbo et al., 2025), reinforcing the value of integrating veterinary diagnostics with targeted entomological surveillance to contextualize such events. In this context, the concurrent detection of WNV RNA in a mosquito pool captured adjacent to the seal enclosure strengthens the inference of local vector-mediated exposure, supporting the local acquisition of infection rather than an imported event. Indeed, genomic and phylogenetic analyses and maximum-likelihood inference placed all sequences within the contemporary southern Italian and regional lineage 2 cluster observed during the 2024–2025 seasons, further supporting local acquisition. Although phylogenetic clustering cannot by itself demonstrate the directionality of transmission, the congruence between host and vector genomes provides a coherent picture for this atypical event and highlights the value of genomics in linking spillover cases to ongoing local viral circulation (Vilibic-Cavlek et al., 2021). Moreover, although the detection of WNV lineage 2 RNA in both harbor seals, together with the identification of infected *Culex pipiens* mosquitoes and the close genomic relatedness of all detected strains, provides strong evidence supporting locally acquired infection, the reasons why only the two seals developed clinical disease cannot be established from the present data. Factors such as enclosure-specific ecological conditions, heterogeneous mosquito biting pressure, behavioral characteristics, or species-specific susceptibility remain plausible, non-mutually exclusive explanations but were not directly investigated in this study. Therefore, these possibilities should be regarded as hypotheses that warrant further ecological, entomological, and host-focused investigations rather than as demonstrated mechanisms underlying the observed cases.

The diagnostic profiles of the two seals were broadly concordant but differed in their immune and virological signals. Seal 1 showed lower Ct values in the brain compared with Seal 2, and WNV RNA was also detected in the heart and spleen, consistent with wider tissue dissemination. In Seal 2, the presence of WNV IgG, together with detectable viral RNA in the brain and spleen, is compatible with infection occurring after seroconversion or with a short overlap between developing humoral immunity and ongoing viral RNA detectability in tissues. Altogether, these patterns suggest that clinically apparent disease may arise at different stages of host response and that viral RNA may remain detectable in multiple organs, even when antibodies are present.

Furthermore, the comparative genomic analysis identified both synonymous and missense substitutions, with amino-acid changes enriched in non-structural proteins (NS1, NS2A, NS3, NS4B, and NS5). These proteins play key roles in viral replication, immune evasion, and host–virus interactions, and variation in these regions has been observed in lineage 2 viruses across outbreaks and host categories (Chancey et al., 2015; Barzon, 2018). In addition, mapping of amino acid changes along the polyprotein shows that non-structural proteins accounted for most of the observed substitutions, a pattern consistent with the central role of these regions in replication and host–virus interactions. In the present cases, some substitutions differed between brain- and spleen-derived genomes, suggesting possible within-host heterogeneity or tissue-associated compartmentalization. However, given the limited number of cases and the absence of functional assays, these patterns should be interpreted cautiously, and observed changes may reflect stochastic drift during infection, bottlenecks between tissues, or sequencing-related thresholds rather than true host adaptation. Nonetheless, documenting such variation in a rare mammalian host contributes valuable baseline data for future comparative studies aimed at distinguishing incidental diversity from signatures potentially associated with replication in non-canonical hosts.

Altogether, these findings extend the known host range of WNV lineage 2 in Europe and underscore the importance of maintaining arbovirus awareness in wildlife during seasonal transmission. Practical implications include strengthening mosquito control, routine entomological surveillance during vector activity, and considering WNV in the differential diagnoses for acute neurological disease in captive mammals, even in species considered atypical or dead-end hosts (Petersen et al., 2013; Chancey et al., 2015; Root and Bosco-Lauth, 2019). From a One Health perspective, zoological settings may serve as early warning systems for local amplification in vectors and wildlife, while genomic characterization can link sentinel events to broader regional dynamics and support risk assessment for both animal and human populations (Riccardo et al., 2022; Vilibic-Cavlek et al., 2021; Gobbo et al., 2025). Zoological parks represent unique ecological interfaces where multiple host species with heterogeneous susceptibility profiles are maintained in proximity under semi-controlled environments. From an epidemiological perspective, these settings create the conditions for investigating the interactions between captive animals, local wildlife reservoirs, and mosquito vectors with an unusually high degree of contextual information. Importantly, zoo grounds are not isolated from the surrounding ecosystem. Free-ranging birds, synanthropic mammals, and mosquitoes can freely move, facilitating the local circulation of pathogens. In this context, zoos may function as sentinel systems, where veterinary diagnostics can be integrated with biosecurity measures, necropsy investigations, and entomological surveillance.

Moreover, zoological parks may offer stable and predictable resources that are often scarce or irregularly distributed in surrounding peri-urban environments. Food provisioning, permanent water availability, and sheltered resting sites can enhance the attractiveness of these areas for synanthropic wildlife and migratory or transient birds, particularly during periods of movement. Reduced predation risk and continuous access to resources may promote longer stopovers and repeated visitation by free-ranging birds, thereby increasing the probability that competent mosquito vectors encounter both reservoir hosts and susceptible captive animals within the same confined setting. Although the extent of this effect likely depends on enclosure design and local biosecurity measures, the peri-urban location of many zoological parks supports the hypothesis that these facilities may function as ecological “patches” in which interactions among wildlife, vectors, and captive species occur more frequently and intensely than in the surrounding urban landscape (Cox-Witton et al., 2014).

From a practical standpoint, these findings support the need for targeted preventive measures in zoological parks during periods of WNV circulation. Such measures may include intensified vector control around enclosures, elimination of standing water and other mosquito breeding sites, routine entomological monitoring, prompt virological investigation of sudden deaths or neurological disease, and proactive surveillance of susceptible co-housed species. Integrating these measures into zoo biosecurity protocols may improve the early detection of arboviral activity and reduce the risk of spillover events in captive animals.

This study also reinforces the need to interpret WNV infections in atypical hosts within a cautious ecological framework. Indeed, the majority of mammals are considered incidental or dead-end hosts, and their epidemiological role is typically limited. Nevertheless, occasional reports of measurable viremia in certain species and episodes of disease in non-canonical hosts indicate that the boundary between spillover and epidemiological relevance may be context-dependent, shaped by host competence, vector abundance, and micro-environmental conditions that modulate contact rates (Root and Bosco-Lauth, 2019).

Future studies should focus on systematic serological screening of co-housed taxa, longitudinal vector sampling, and expanded genomic analysis across tissues and hosts to better define the frequency and epidemiological relevance of WNV infection in marine mammals under captive conditions.

Finally, although this study provides strong virological, entomological, and genomic evidence supporting WNV lineage 2 infection in two captive harbor seals, some limitations should be considered when interpreting the findings: (i) the report is based on only two cases, which is inherently insufficient to infer broader host-level or population-level patterns; (ii) histopathological examination was available for only one animal, limiting direct clinicopathological comparison between the two seals. Furthermore, from a clinical and pathological perspective, the interpretation of these cases is limited by the relatively sparse characterization of some extra-neural tissues and by the absence of a full systematic microscopic assessment across all organs. Moreover, while the central nervous system lesions were compatible with WNV neuroinvasion, a broader histopathological survey of additional organs would have further clarified the extent of systemic involvement. These limitations should be considered when interpreting the clinicopathological spectrum of infection in these animals; and (iii) some interpretations regarding host susceptibility, enclosure microecology, and mosquito exposure remain speculative and should be regarded as hypotheses rather than demonstrated mechanisms. Finally, the inclusion of immunohistochemistry and/or *in situ* hybridization would have further strengthened the demonstration of viral localization within the affected tissues, particularly in the central nervous system; however, these techniques were not available in the present investigation. Despite these limitations, the concordance of clinical, molecular, entomological, and phylogenetic findings supports the interpretation of a locally acquired WNV infection associated with fatal neuroinvasive disease.

## Data Availability

The data presented in this study are deposited in the NCBI repository, accession numbers PZ143793 (mosquito pool), PZ143802 (seal 1), and PZ143798 (seal 2). Other data supporting the findings of this study are available within the article and its Supplementary material.

## Group member of WNV One Health Surveillance Network

The WNV One Health Network is a multidisciplinary collaborative network supporting West Nile virus surveillance, diagnosis, genomic investigation, and One Health response activities; Marco Campolo, Parco Zoo Falconara, Ancona, Italy; Lo Zoo di Napoli, Naples, Italy; Daniela Izzillo, Azienda Sanitaria Locale Napoli 1 Centro, Naples, Italy; Luigi De Luca Bossa, Azienda Sanitaria Locale Napoli 1 Centro, Naples, Italy; Riccardo Romanucci, Azienda Sanitaria Locale Napoli 1 Centro, Naples, Italy;Guido Rosato, Azienda Sanitaria Locale Napoli 1 Centro, Naples, Italy; Marina Pompameo, Azienda Sanitaria Locale Napoli 1 Centro, Naples, Italy; Marialuisa Buonanno, Istituto Zooprofilattico Sperimentale del Mezzogiorno, Portici, Naples, Italy; Loredana Baldi, Istituto Zooprofilattico Sperimentale del Mezzogiorno, Portici, Naples, Italy;Francescantonio D’Orilia, Azienda Sanitaria Locale Salerno, Salerno, Italy; Daniela Morelli, Istituto Zooprofilattico Sperimentale dell'Abruzzo e del Molise "G. Caporale", Teramo, Italy; Marco Esposito, U.O.D. Prevention and Public Health, Campania Region, Naples, Italy; Celestina Mascolo, General Directorate for Health Protection and Coordination of the Regional Health System-Unit of Prevention and Veterinary Public Health, Naples, Italy; Michele Valentino Chiara, Health Protection and Coordination of the Regional Health System. Social and Health Policies, Campania Region, Naples, Italy.
