# Supplementary material for: Detection and genomic characterization of West Nile virus lineage 2 in captive harbor seals in southern Italy
Source: Front Microbiol. 2026 Sep 16;17:1937304. doi: 10.3389/fmicb.2026.1937304 (PMC13624066; doi:10.3389/fmicb.2026.1937304)
Supplement: Supplementary file 1 [file Table_1.docx]

**Supplementary materials**

**Table 1.  Protocols used for virological, bacterial, and protozoan investigations.**

| **Organs** | **Pathogen** | **Primer and Probe Sequence (5′- 3′)** | **Reference** |
| --- | --- | --- | --- |
| Brain, heart, spleen, kidneys | West Nile disease | **Primers**  WN-LCV-F1- 5'-GTGATCCATGTAAGCCCTCAGAA-3' PFT  WN-LCV-R1 5'-GTCTGACATTGGGCTTTGAAGTTA-3' PRT  **Probes**  WN-LCV-S1 FAM-5'-AGGACCCCACATGTT-3'-MGB ST1  WN-LCV-S2 VIC-5'-AGGACCCCACGTGCT-3'-MGB ST2 | Del Amo J, Sotelo E, Fernandez-Pinero J, et al. A novel quantitative multiplex real-time RT-PCR for the simultaneous detection and differentiation of West Nile virus lineage 1 e 2, and of Usutu virus. *J Virol Methods* 2013;189 (2):321-327. |
| Brain, heart, spleen, kidneys | Usutu virus | **Primers**  USU-For   5-AAAAATGTACGCGGATGACACA-3'  USU-Rew 5'TTTGGCCTCGTTGTCAAGATC-3'  **Probe**  USU-P FAM-5'-CGGCTGGGACACCCGGATAACC-TAMRA-3' | Cavrini F, Della Pepa ME, et al. A rapid and specific real-time RT-PCR assay to identify Usutu virus in human plasma, serum, and cerebrospinal fluid. *J Clin Virol* 2011;50:221-3. |
| Brain, lungs, trachea | Avian flu | **Primers**  IVA-D161M 5’-AGATGAGYCTTCTAACCGAGGTCG-3’  IVA-D162M1   5’-TGCAAAAACATCYTCAAGTCTCTG-3’  IVA-D162M2   5’-TGCAAACACATCYTCAAGTCTCTG-3’  IVA-D163M3   5’-TGCAAAGACATCYTCAAGTCTCTG-3’  IVA-D162M4   5’-TGCAAATACATCYTCAAGTCTCTG-3’  **Probe**  IVA MA  FAM-5’-TCAGGCCCCCTCAAAGCCGA-3’-TAMRA | Heine HG, Foord AJ, Wang J, et al. Detection of highly pathogenic zoonotic influenza virus H5N6 by reverse-transcriptase quantitative polymerase chain reaction. *Virol J* 2015;12:18. |
| Brain, lungs | Canine  distemper  virus (CDV) | **Primers**  CDV F-5’- AGCTAGTTTCATCTTAACTATCAAATT -3’  CDV R-5’- TTAACTCTCCAGAAAACTCATGC -3’  **Probe**  CDV P-FAM-5'- ACCCAAGAGCCGGATACATAGTTTCAATGC- 3’-TAMRA | Elia G, Decaro N, Martella V, et al. Detection of canine distemper virus in dogs by real-time RT-PCR. *J Virol Methods* 2006;136(1-2):171-6. |
| Brain, heart | Neospora - Toxoplasma | **Primers**  APIF   5’ AAGTATAAGCTTTTATACGGC-3’  APIR   5’ CACTGCCACGGTAGTCCAATAC-3’ | Santoro M, D'Alessio N, Cerrone A, et al. The Eurasian otter (Lutra lutra) as a potential host for rickettsial pathogens in southern Italy. PLoS One. 2017 Mar 7;12(3):e0173556. |
| Kidneys | *Leptospira* spp. | **Primers**  LipL32-45F 5’-AAGCATTACCGCTTGTGGTG-3’  LipL32-286R 5’-GAACTCCCATTTCAGCGATT-3’  **Probe**  LipL32-189P FAM-5′-AA AGC CAG GAC AAG CGC CG-3′-BHQ1 | Stoddard RA, Gee JE, Wilkins PP, et al. Detection of pathogenic Leptospira spp. through TaqMan polymerase chain reaction targeting the LipL32 gene. *Diagn Microbiol Infect Dis* 2009;64(3):247-55. |
| Submandibular lymph nodes | *Mycobacterium tuberculosis complex* | **Primers**  EXT-1 5′-CCCGGACAGGCCGAGTTT-3′  INT-1 5′-CCCCATCGACCTACTACG-3′  **Probe**  IS6110 5′-FAM-AACTCAAGGAGCAGTCAGGCH-BHQ1-3′ | Vangone L, Cardillo L, Riccardi MG, et al. Mycobacterium tuberculosis SIT42 Infection in an Abused Dog in Southern Italy. *Front Vet Sci* 2021;8:653360. |
| Testis | *Brucella spp* | Bacterial isolation | World Organisation for Aniaml Health (WOAH). Brucellosis (infection with Brucella abortus, B. melitensis and B. suis). Chapter 3.1.4. Most recent updates adopted in 2022. Available form: <https://www.woah.org/en/what-we-do/standards/codes-and-manuals/> [accessed on 21 May 2026]. |

**Seal 1: complete blood count**

| **Parameter** | **Results** | **Unit** | **Reference Interval**  **(*P. vitulina*)** |
| --- | --- | --- | --- |
| **Hematocrit (HCT)** | 31.2 | % | 35.0–78.0 |
| **Hemoglobin (HGB)** | 11.6 | g/dL | 9.0–35.2 |
| **Erythrocytes (RBC)** | 3.24 | ×10⁶/mm³ | 2.98–7.11 |
| **Leukocytes (WBC)** | 7.84 | ×10³/mm³ | 3.08–25.80 |
| **Corrected Leukocytes** | 7.84 | ×10³/mm³ | — |
| **Neutrophils** | 3.53 | ×10³/mm³ | 1.45–19.6 |
| **Band Neutrophils** | 0.00 | ×10³/mm³ | 0.031–1.510 |
| **Lymphocytes** | 1.65 | ×10³/mm³ | 0.360–6.520 |
| **Eosinophils** | 0.00 | ×10³/mm³ | 0.010–1.733 |
| **Monocytes** | 2.57 | ×10³/mm³ | 0.047–3.354 |
| **Basophils** | 0.14 | ×10³/mm³ | 0.005–0.633 |
| **Undifferentiated Cells** | 0.00 | ×10³/mm³ | — |
| **Reticulocytes** | — | % | — |
| **Reticulocyte Index** | — | — | — |
| **RDW** | 14.8 | % | — |
| **Platelets (PLT)** | 489 | ×10³/µL | 99–507 |
| **Platelet Estimate** | Increased | — | — |
| **MPV (Mean Platelet Volume)** | 7.8 | fL | — |
| **MCV (Mean Corpuscular Volume)** | 96.3 | fL | 61.5–177.9 |
| **MCH (Mean Corpuscular Hemoglobin)** | 35.8 | pg | 23.4–88.0 |
| **MCHC (Mean Corpuscular Hemoglobin Concentration)** | 37.2 | g/dL | 30.9–69.0 |

**Seal 1: biochemical analysis**

| **Parameter** | **Results** | **Unit** | **Reference interval**  ***(P. vitulina*)** |
| --- | --- | --- | --- |
| **Urea** | 110 | mg/dl | 42.8–190.4 |
| **Creatinine** | 1.14 | mg/dl | 0.6–2.8 |
| **Glucose** | 133 | mg/dl | 75–230 |
| **Fructosamine** | — | µmol/l | — |
| **Total Protein** | 8.01 | g/dl | 5.2–10.6 |
| **Albumin** | 3.5 | g/dl | 2.8–6.0 |
| **Globulin** | 4.51 | g/dl | 2.1–6.9 |
| **Albumin/Globulin Ratio** | 0.8 | — | — |
| **Bilirubin** | 0.79 | mg/dl | 0.0–1.3 |
| **Aspartate Aminotransferase (AST)** | 441 | U/l at 25°C | 20–237 |
| **Alanine Aminotransferase (ALT)** | 146 | U/l at 25°C | 5–254 |
| **Gamma-Glutamyl Transferase (GGT)** | 15.9 | U/l at 25°C | 0–62 |
| **Alkaline Phosphatase (ALP)** | 47 | U/l at 25°C | 9–218 |
| **Bicarbonate** | 14.67 | mmol/l | 19.0–28.0 |
| **Lactate** | 118 | mg/dl | — |
| **Amylase** | 1235 | U/l at 25°C | 337–1426 |
| **Lactate Dehydrogenase (LDH)** | 1956 | U/l at 25°C | 131–1728 |
| **Creatine Phosphokinase (CPK)** | 751 | U/l at 25°C | 4–1687 |
| **Total Cholesterol** | 254 | mg/dl | 168–439 |
| **Triglycerides** | 55 | mg/dl | 22–196 |
| **Total Iron** | 158 | µg/dl | 55–234 |
| **Magnesium** | 1.91 | mg/dl | 1.10–2.67 |
| **Chloride** | 109.1 | mEq/l | 83–128 |
| **Corrected Chloride** | 110.8 | mEq/l | 83–128 |
| **Sodium** | 143.8 | mEq/l | 132–167 |
| **Potassium** | 3.17 | mEq/l | 3.0–6.0 |
| **Sodium/Potassium Ratio** | 45.4 | — | — |
| **Ionized Calcium** | 1.09 | mmol/l | — |
| **pH** | 7.17 | — | — |
| **Calcium** | 8.1 | mg/dl | 7.5–11.8 |
| **Phosphorus** | 6.8 | mg/dl | 1.8–10.5 |
| **Calcium × Phosphorus Product** | 55.08 | — | — |
| **Anion Gap** | 23.2 | — | — |
| **Estimated Osmolality** | 329.0 | mOsm/kg | 316–326 |

**Seal 1: serum protein electrophoresis**

**Seal 2: complete blood count test**

| **Parameter** | **Result** | **Unit** | **Reference Interval**  **(*P. vitulina*)** |
| --- | --- | --- | --- |
| **Hematocrit (HCT)** | 27 | % | 35.0–78.0 |
| **Hemoglobin (HGB)** | 9.37 | g/dL | 9.0–35.2 |
| **Erythrocytes (RBC)** | 2.75 | ×10⁶/mm³ | 2.98–7.11 |
| **Leukocytes (WBC)** | 4.44 | ×10³/mm³ | 3.08–25.80 |
| **Corrected Leukocytes** | 4.44 | ×10³/mm³ | — |
| **Neutrophils** | 2.49 | ×10³/mm³ | 1.45–19.6 |
| **Band Neutrophils** | 0.18 | ×10³/mm³ | 0.031–1.510 |
| **Lymphocytes** | 0.63 | ×10³/mm³ | 0.360–6.520 |
| **Eosinophils** | 0.03 | ×10³/mm³ | 0.010–1.733 |
| **Monocytes** | 1.16 | ×10³/mm³ | 0.047–3.354 |
| **Basophils** | 0.00 | ×10³/mm³ | 0.005–0.633 |
| **Undifferentiated Cells** | 0.00 | ×10³/mm³ | — |
| **Reticulocytes** | — | % | — |
| **Reticulocyte Index** | — | — | — |
| **RDW** | 13.3 | % | — |
| **Platelets (PLT)** | 471 | ×10³/µL | 99–507 |
| **Platelet Estimate** | Increased | — | — |
| **MPV (Mean Platelet Volume)** | 7.43 | fL | — |
| **MCV (Mean Corpuscular Volume)** | 98.2 | fL | 61.5–177.9 |
| **MCH (Mean Corpuscular Hemoglobin)** | 34.1 | pg | 23.4–88.0 |
| **MCHC (Mean Corpuscular Hemoglobin Concentration)** | 34.7 | g/dL | 30.9–69.0 |

**Seal 2: biochemical analyses**

| **Parameter** | **Result** | **Unit** | **Reference Interval (*P. vitulina*)** |
| --- | --- | --- | --- |
| **Urea** | 31 | mg/dL | 42.8–90.4 |
| **Creatinine** | 0.65 | mg/dL | 0.6–2.8 |
| **Glucose** | 101 | mg/dL | 75–230 |
| **Fructosamine** | — | µmol/L | — |
| **Total Proteins** | 8.8 | g/dL | 5.2–10.6 |
| **Albumin** | 4.1 | g/dL | 2.8–6.0 |
| **Globulins** | 4.7 | g/dL | 2.1–6.9 |
| **Albumin/Globulin Ratio** | 0.9 | — | — |
| **Bilirubin** | 0.33 | mg/dL | 0.0–1.3 |
| **AST** | 123 | U/L at 25°C | 20–237 |
| **ALT** | 75 | U/L at 25°C | 5–254 |
| **GGT** | 11.7 | U/L at 25°C | 0–62 |
| **ALP** | 8 | U/L at 25°C | 9–218 |
| **Bicarbonate** | 15.78 | mmol/L | 19.0–28.0 |
| **Lactate** | 131.9 | mg/dL | — |
| **Amylase** | 405 | U/L at 25°C | 337–1426 |
| **LDH** | 1199 | U/L at 25°C | 131–1728 |
| **CPK** | 238 | U/L at 25°C | 4–687 |
| **Total Cholesterol** | 239 | mg/dL | 168–439 |
| **Triglycerides** | 39 | mg/dL | 22–196 |
| **Serum Iron** | 55 | µg/dL | 55–234 |
| **Magnesium** | 0.49 | mg/dL | 1.10–2.67 |
| **Chloride** | 102.2 | mEq/L | 83–128 |
| **Corrected Chloride** | 104.2 | mEq/L | 83–128 |
| **Sodium** | 143.2 | mEq/L | 132–167 |
| **Potassium** | 4.94 | mEq/L | 3.0–6.0 |
| **Na/K Ratio** | 29.0 | — | — |
| **Calcium (Ca++)** | 0.92 | mmol/L | — |
| **pH** | 7.21 | — | — |
| **Ionized Calcium** | 8.3 | mg/dL | 7.5–11.8 |
| **Phosphorus** | 5.1 | mg/dL | 1.8–10.5 |
| **Calcium × Phosphorus Product** | 42.33 | mg/dL | — |
| **Anion Gap** | 30.16 | mEq/L | — |
| **Estimated Osmolality** | 301.2 | mOsm/kg | 316–326 |

- **Hemolysis**: positive (+)
- **Lipemia**: negative
- **Icterus**: positive (+)

**Seal 2: serum protein electrophoresis**
